# Blood Immunophenotyping in Prediction of Gestational Hypertensive Conditions

**DOI:** 10.3390/biomedicines13123122

**Published:** 2025-12-18

**Authors:** Almagul Kurmanova, Altynay Nurmakova, Damilya Salimbayeva, Gulfiruz Urazbayeva, Gaukhar Kurmanova, Natalya Kravtsova, Zhanar Kypshakbayeva, Madina Khalmirzaeva

**Affiliations:** 1 Faculty of Medicine and Healthcare, Al-Farabi Kazakh National University, 71 Al-Farabi Ave., Almaty 050040, Kazakhstanmadinakhalmirzaeva7@gmail.com (M.K.); 2 Department of Strategic Development and Science, Scientific Center for Obstetrics, Gynecology and Perinatology, 125 Dostyk Ave., Almaty 050010, Kazakhstan; sdamilya@mail.ru (D.S.);; 3 Department of Obstetrics and Gynecology with a Course in Clinical Genetics, S.D. Asfendiyarov Kazakh National Medical University, 94 Tole Bi Ave., Almaty 050012, Kazakhstan

**Keywords:** hypertensive conditions, preeclampsia, CD-phenotyping, cytokines, IL-10, TNF, GM-CSF, IGF

## Abstract

**Background**: Hypertensive conditions during pregnancy, such as preeclampsia (PE), are multisystem obstetric complications, accompanied by changes in the immunological status. Although several types of immune cells are involved in pathogenesis of preeclampsia, such as regulatory T cells, macrophages, natural killer cells, and neutrophils, most studies have focused on the concentration of circulating cytokines. Much less is known about intracellular cytokine production at the level of individual groups of peripheral blood immune cells. This gap limits our understanding of the early immunological changes that precede the clinical manifestation of the disease. Thus, the study of intracellular cytokine production in various leukocyte populations may provide new biomarkers for predicting preeclampsia. **Objectives**: To test the hypothesis that women with preeclampsia exhibit distinct intracellular cytokine profiles in specific peripheral blood immune cell subsets compared with normotensive pregnant women, and to assess whether these differences could serve as potential biomarkers for disease prediction. **Methods**: The study included a total of 78 pregnant women admitted to labor with physiological pregnancy (n = 32) and with gestational hypertension (GH) (n = 39) and PE (n = 7). The multicolor immunophenotyping with intracellular cytokine production of TNF, GM-CSF, IGF and receptor VEGFR-2 by different immunocompetent cell types was evaluated on a BD FACS CALIBUR flow cytometer. **Results**: Flow cytometry revealed a marked increase in the proportion of CD8^+^ GM-CSF^+^, CD56^+^VEGFR2^+^, CD14^+^IL-10^+^, and CD19^+^IGF^+^ cells in both hypertensive groups versus controls (*p* < 0.001). In contrast, CD56^+^TNF^+^ levels were significantly reduced (*p* < 0.001). For differentiating PE from GH, CD56+VEGFR2+ and CD19+IGF+ should be prioritized (AUC~0.66–0.78) with good specificity and moderate sensitivity. **Conclusions**: These data will not only expand existing knowledge about the role of intracellular cytokines in the pathogenesis of preeclampsia, but will also help to obtain new markers for predicting preeclampsia.

## 1. Introduction

Hypertensive conditions during pregnancy, including preeclampsia (PE), are a multisystem obstetric complication that contributes significantly to maternal and neonatal mortality. In Kazakhstan, PE ranks second in the structure of maternal mortality, accounting for 11% of all obstetric causes. According to the International Society for the Study of Hypertension in Pregnancy (ISSHP), preeclampsia (de novo) is a hypertensive condition (systolic blood pressure (BP) ≥ 140 mmHg, diastolic BP ≥ 90 mmHg), accompanied by one or more new complications after 20 weeks of pregnancy: proteinuria, neurological complications, renal and hepatic damage, hematological complications, pulmonary edema, uteroplacental dysfunction [1]. There is also evidence that women who have had preeclampsia have an increased risk of developing cardiovascular complications throughout their lives [2]. Currently, it is believed that immune regulation disorders and endothelial dysfunction are involved in the development of the disease, but the exact molecular mechanisms remain unclear, and reliable methods for early prediction of preeclampsia are lacking [3,4]. According to the literature, preeclampsia is divided into early and late PE, but these terms are not yet officially used in clinical protocols. Early PE occurs before 34 weeks of pregnancy and accounts for approximately 5–20% of all cases of PE worldwide, and is also characterized by greater danger to both the mother and the fetus. Late PE occurs after 34 weeks of pregnancy and accounts for 80–95% of cases worldwide [5,6]. The mechanisms of early and late development of PE may not be entirely the same. It is known that the pathogenesis of PE is associated with reduced blood supply to the placenta, which in turn leads to fetal growth disorders and increases the risk of stillbirth. It is believed that preeclampsia develops in two phases: (1) abnormal placentation in the first trimester, followed by (2) “maternal syndrome” in the late second and third trimesters, which is characterized by an excess of angiogenic factors [7].

Despite intensive research, the pathogenesis of PE remains poorly understood, and universal biomarkers for early prediction and diagnosis have not yet been approved in international guidelines. In recent years, particular attention has been paid to immunological mechanisms, in particular, immune disorders are considered a key link: there is a shift towards Th1/Th17 (pro-inflammatory cytokines TNF-α, IL-17) and a decrease in the Th2/Treg response (IL-10, IL-4), creating an unproductive, persistent inflammatory background [8]. Studies show that TNF-α is a key pro-inflammatory cytokine that plays a central role in the pathogenesis of preeclampsia. A review by LaMarca et al. emphasizes that preeclampsia activates innate and adaptive immune cells and increases TNF-α production, leading to endothelial dysfunction, vasoconstriction, and hypertension [9].

Unlike TNF-α, IL-10 is a key anti-inflammatory cytokine that maintains immune tolerance during pregnancy. The study of Salvany-Celades et al. demonstrated the existence of several functional subtypes of regulatory T cells (Treg) at the maternal–fetal interface, which suppress effector T cell responses through IL-10 production, ensuring immune balance and preventing excessive inflammation [10].

The role of granulocyte-macrophage colony-stimulating factor (GM-CSF) in PE remains a subject of debate. A number of studies have shown increased levels of GM-CSF in both peripheral blood and placental tissue in women with PE, indicating its possible involvement in immune dysregulation and the formation of an inflammatory response [11,12]. However, our recent study showed a decrease in GM-CSF expression in the placenta in PE [13], which is consistent with the data of Tian et al., where it was shown that a decrease in PD-L1 in trophoblasts is associated with the suppression of GM-CSF through the activation of the JAK2/STAT5 pathway; an increase in PD-L1 in a PE-like model in rats partially alleviated hypertension and proteinuria [14].

Insulin-like growth factor 1 (IGF-1) is associated with cell growth, metabolism, angiogenesis, and differentiation [15]. One study showed that IGF-1 may inhibit the development of preeclampsia by decreasing miR-183 expression by increasing ZEB1 expression. Other molecular mechanisms may also contribute to improved prediction of preeclampsia and suggests new promising therapeutic targets for the treatment of PE [16]. The impact of the COVID-19 pandemic on changes in immunoreactivity, including during pregnancy, cannot be ruled out. Thus, in pregnant women with SARS-CoV-2 infection, the risk of developing preeclampsia was significantly higher than in women without infection [8].

This study is a follow-up to a recent article published in the journal Diagnostics (2025) [13], which compared immunological parameters in peripheral blood and placental tissue in preeclampsia and healthy pregnant women. Unlike the previous article, this study compares intracellular markers between patients with gestational hypertension, preeclampsia, and healthy pregnant women [13]. We hypothesize that dysregulation of intracellular cytokine production in peripheral immune cells contributes to the development of gestational hypertension and preeclampsia. Specifically, altered expression of pro-inflammatory (TNF, GM-CSF) and angiogenic or growth-related markers (IGF, IL-10) reflects an imbalance between inflammatory activation and compensatory repair mechanisms at the maternal–fetal interface. This imbalance may differentiate physiological adaptation in normal pregnancy from maladaptive responses observed in hypertensive disorders of pregnancy.

## 2. Materials and Methods

### 2.1. Study Design and Setting

This was a prospective cross-sectional observational study conducted at the Scientific Center for Obstetrics, Gynecology and Perinatology (Almaty, Kazakhstan). Recruitment took place during routine hospital admissions. Peripheral blood samples and clinical data were collected at the time of admission, prior to delivery.

### 2.2. Participants and Recruitment Process

Participants were identified consecutively upon admission either to the Department of Pregnancy Pathology or to the Delivery Unit. Women were then assigned to one of three study groups based on clinical diagnosis: Gestational hypertension (GH), Preeclampsia (PE), Control (uncomplicated pregnancy). Diagnosis of GH and PE was established and independently verified by two obstetricians using ISSHP diagnostic criteria (blood pressure measurements, proteinuria, and signs of organ dysfunction based on medical records).

The control group was formed using a convenience sampling approach from women with uncomplicated pregnancies who delivered during the same recruitment period. No matching procedures (e.g., by age or anthropometry) were applied, reflecting real-world admission flow.

Inclusion criteria for gestational hypertension group: pregnant women aged 18 years and older, with a new onset of high blood pressure (systolic ≥140 mmHg or diastolic ≥90 mmHg) after 20 weeks of pregnancy who previously had normal blood pressure, the absence of proteinuria or other signs of organ damage.

Inclusion criteria for in the PE group: pregnant women aged 18 years and older, blood pressure >140/90 mm Hg after 20 weeks, proteinuria >0.3 g/day, as well as the presence of anamnestic, laboratory, and instrumental signs and clinical manifestations of organ damage against the background of hypertension.

Inclusion criteria for the control group: pregnant women over 18 years of age, pregnancy not complicated by hypertension.

Exclusion criteria for both groups: acute and chronic inflammatory diseases, severe extragenital pathology, previous organ transplantation, previous cancer, diabetes mellitus, blood transfusion, systemic autoimmune diseases, tuberculosis, HIV, chronic arterial hypertension. All patients signed an informed consent form to participate in the study.

A STROBE-compliant participant flow diagram is provided in the Supplementary Materials (Figure S1).

The groups were comparable in age and atropometric data; when studying clinical and anamnestic data (family history of preeclampsia in the mother or sister, presence of allergies, anemia, previous COVID-19), no statistical differences were revealed.

### 2.3. Ethical Approval

The work was carried out in accordance with the principles of voluntariness and confidentiality based on the Helsinki Declaration. The study was approved by the Local Ethics Committee of the Scientific Center for Obstetrics, Gynecology, and Perinatology (No. 2 dated 9 November 2022). All participants gave written informed consent for the use of biomaterials in this study. The material was anonymized.

### 2.4. Sample Processing

The peripheral blood in a volume in a standard K2EDTA/K3EDTA tube was collected immediately and transferred to the Immunology Laboratory of the Scientific Center for Obstetrics, Gynecology and Perinatology (Almaty, Kazakhstan).

### 2.5. Immunophenotyping

The blood was conducted according to the manufacturer’s protocol (www.bdbiosciences.com). The sample was treated with a protein transport inhibitor (contains monensin) BD GolgiStop™ (Becton Dickinson Hungary Kft., Környe, Hungary), resuspended and transferred to plastic tubes for immunofluorescent staining, 50 µL of the blood sample was incubated with 5 µL of fluorochrome-conjugated monoclonal antibodies (mAb) for surface marker analysis for 15 min at room temperature in the dark. The samples were stained with monoclonal antibodies (mAb) using Becton Dickinson (BD) conjugated with fluorescein isothyocyanate (FITC) reagents against the surface receptors CD4, CD8, CD56, CD14, and CD19. After staining, erythrocytes were lysed using BD FACS™ Lysing Solution (Becton Dickinson Hungary Kft.), incubated for 10 min in the dark at room temperature, and centrifuged at 300× *g* for 5 min. The supernatant was removed, resuspend cells were processed with Cytofix/Cytoperm™ (Becton Dickinson Hungary Kft.) Plus Fixation/Permeabilization Kit, followed by mAb conjugated with PerCP-Cy5.5 or Phycoerythrin (PE) for staining and binding intracellular receptors against TNF, IL-10, GM-CSF, VEGFR-2 and IGF.

### 2.6. Flow Cytometric Analysis

The gating strategy was based on FSC/SSC size and granularity. Lymphocytes were smaller and had low granularity, while monocytes were larger and more granular. The next step involved gating based on marker expression: CD45+ and CD14+ (monocytes) and CD45+ and CD14− (lymphocytes). The total population of leukocyte cells was isolated using the CD45+ marker, then lymphocytes and monocytes were isolated from this fraction. Concentration-matched isotype controls were used to set the gates and single-fluorochrome stained controls were used to compensate for spectral overlap. The immunophenotyping with cytokine production of cells were evaluated on a BD FACS CALIBUR flow cytometer (New York, NY, USA) and the data was analyzed using the CELL Quest program.

### 2.7. Statistical Analysis

The normality of the distribution of quantitative variables was assessed using the Shapiro–Wilk test. The homogeneity of the variances was checked by the Levene criterion. For variables with normally distributed values but heterogeneous variances, the Welch one-way ANOVA was used, and paired multiple comparisons were performed using the Games–Howell test, which is resistant to inequality of variances and group sizes. For variables with a deviation from normality (for example, CD14^+^/IL-10^+^), the nonparametric Kruskal–Wallis test was used, followed by pairwise comparisons according to Dunn with the Bonferroni adjustment. The statistical significance of differences in quantitative data between groups was calculated using the Mann–Whitney U test. To compare the characteristics of the studied groups, due to the small sample, the Fisher criterion was used. To assess the accuracy of the estimates, confidence intervals (CI) were calculated for clinical variables. The *p* value < 0.05 was considered statistically significant. All statistical calculations were performed in SPSS program (Version 27, available online: IBM SPSS Software, https://www.ibm.com/products/spss (accessed on 9 December 2025)). The characteristics of the markers were evaluated via ROC analysis using the tool Microsoft Copilot in Python version 3.10.18.

## 3. Results

### 3.1. Clinical Data

The study included 39 women with gestational hypertension, 7 women with preeclampsia and 32 women from a control group matched for age and anthropometric parameters (Table 1).

The mean age of patients with gestational hypertension (GH) was 34.5 ± 6.8 years, in preeclampsia (PE)—33.5 ± 3.86 years, and in the control group—33.3 ± 6.39 years, showing no significant difference between the groups (*p* = 0.665). Body weight also did not differ significantly: 86.6 ± 18.3 kg in GH, 82.1 ± 9.37 kg in PE, and 81.3 ± 14.9 kg in the control group (*p* = 0.06). Height values were comparable as well: 158.2 ± 24.4 cm, 166 ± 1.5 cm, and 163 ± 5.7 cm, respectively (*p* = 0.109).

In contrast, hemodynamic parameters demonstrated statistically significant differences between the groups. Systolic blood pressure was markedly higher in women with GH (142 ± 1.8 mmHg) and PE (140 ± 11 mmHg) compared to the control group (102.9 ± 9.57 mmHg; *p* < 0.001). Similarly, diastolic blood pressure was significantly elevated in GH (90.4 ± 7.2 mmHg) and PE (84.3 ± 7.87 mmHg) compared to controls (67.5 ± 12.4 mmHg; *p* < 0.001).

The mean gestational age at diagnosis for PE was 36.2 ± 2.14 weeks, slightly higher than in GH (30.7 ± 6.7 weeks; *p* = 0.06).

Total gestational duration was significantly shorter in GH (38.5 ± 0.8 weeks) and PE (36.5 ± 4 weeks) compared to the control group (39.5 ± 1.21 weeks; *p* < 0.001).

These findings were also reflected in perinatal outcomes: the mean neonatal weight was 3428 ± 475 g in GH, 3041 ± 672 g in PE, and 3600 ± 420 g in the control group (*p* = 0.05).

The clinical profile of pregnant women with gestational hypertension and preeclampsia was characterized by a significant increase in both systolic and diastolic blood pressure compared to healthy controls. Despite similar maternal age, body weight, and height across the groups, women with hypertensive disorders had shorter gestational periods and delivered infants with lower birth weight. These findings indicate that, even in the absence of pronounced anthropometric differences, hypertensive complications in pregnancy are associated with adverse perinatal outcomes and reflect a distinct clinical pattern of maternal hemodynamic imbalance.

### 3.2. Immunological Parameters in Peripheral Blood

The study analyzed the intracellular cytokine profiles of the main immune cell subpopulations isolated from the peripheral blood of women with GH, preeclampsia and a control group with physiological pregnancies. Double phenotypes were used for evaluation: CD8^+^GM-CSF^+^, CD56^+^TNF^+^, and CD14^+^IL-10^+^. Immunological parameters in peripheral blood are shown in Table 2.

The verification of the distribution of quantitative data was performed by the Shapiro–Wilk criterion. Most of the parameters studied had a normal distribution (skewness and kurtosis within ±1); the data are presented as a mean and an average with a 95% confidence interval. For signs with deviation from normality (CD56^+^TNF^+^ and CD14^+^IL-10^+^), the median and interquartile range, IQR (interquartile range), were used, and nonparametric Mann—Whitney and Kraskel–Wallis tests were used for statistical analysis.

The content of CD8^+^GM-CSF^+^ cells was significantly increased in both hypertension and preeclampsia compared with the control (29.97; 95% CI 28.77–31.01 and 28.58; 95% CI 25.04–31.9 vs. 7.33; 95% CI 6.74–7.83), with differences between hypertension and preeclampsia No overlapping 95% CI was detected.

The average CD56^+^TNF^+^ lymphocyte count in the control group (15.95; IQR 1.84) was significantly higher than in women with gestational hypertension (1.42; IQR 1.06) and preeclampsia (2.19; IQR 2.31). The overlap of confidence intervals between hypertension and preeclampsia indicates that there are no significant differences between them; however, both groups are characterized by a marked decrease in TNF-producing NK cells compared with normal pregnancy. The results obtained indicate suppression of the innate immune link in hypertensive pregnancy complications and impaired cytokine-mediated regulation of the placental immune microclimate.

The average level of CD56^+^VEGF2^+^ cells increased sequentially from physiological pregnancy to gestational hypertension and further to preeclampsia: 0.55 (95% CI 0.39–0.78) → 10.53 (95% CI 9.97–11.28) → 14.2 (95% CI 11.02–17.43). The confidence intervals did not overlap, which indicates a significant increase in angiogenic activity of NK cells as the pathological process worsened. The data obtained indicate a compensatory and then pathological increase in angiogenesis in hypertensive complications of pregnancy, reflecting a violation of the immune–angiogenic interaction in the placental system.

The average level of CD14^+^IL-10^+^ cells consistently differed between the groups: control—0.77; IQR 1.09, hypertension—68.65; IQR 7.52 and preeclampsia—55.68; IQR 31.02. The differences between all three groups are statistically significant according to non-overlapping confidence intervals. The results obtained reflect the phasic nature of the anti-inflammatory response: compensatory enhancement of IL-10 production in gestational hypertension and its depletion in preeclampsia, which confirms the role of monocyte-regulatory immunity in the pathogenesis of preeclampsia.

The average CD19^+^IGF^+^ lymphocyte count in pregnant women with gestational hypertension (9.68; 95% CI 8.98–10.39) and preeclampsia (12.16; 95% CI 8.34–15.72) was significantly higher than in the control group (1.08; 95% CI 0.94–1.26). The absence of overlapping confidence intervals between the control and the pathological groups confirms the validity of the differences. The transition from hypertension to preeclampsia is accompanied by a tendency to further increase CD19^+^IGF^+^ cells and an increase in the spread of values, reflecting a violation of humoral-angiogenic regulation. The data obtained confirm the involvement of the B-cell link in the pathogenesis of preeclampsia through the expression of placental growth factors.

### 3.3. Statistical Analysis for Immunological Parameters in Peripheral Blood

To assess the differences between the three groups (control, gestational hypertension, preeclampsia), a one-way analysis of variance (ANOVA) was performed. Statistically significant differences between the groups were found for all markers studied (*p* < 0.001) in Table 3 and Table 4.

A comparative analysis was performed of the levels of CD8^+^GM-CSF^+^, CD56^+^VEGFR2^+^, and CD19^+^IGF^+^ immune subpopulations in pregnant women in the control group, with gestational hypertension, and with preeclampsia. The mean values of all three indicators were significantly higher in patients with hypertension and preeclampsia compared to the control group (*p* < 0.001). Thus, the level of CD8^+^GM-CSF^+^ cells in hypertension was 29.97 ± 3.4, in PE—28.58 ± 3.7 versus 7.3 ± 1.5 in the control. Similar trends were observed for CD56^+^VEGFR2^+^ (10.6–14.2 versus 0.6) and CD19^+^IGF^+^ (9.7–12.0 versus 1.1).

According to the Levene’s criterion, heterogeneity of variances was detected (*p* < 0.05), therefore the Games–Howell test was used for multiple comparisons. A posteriori analysis confirmed significant differences between the control and both pathological groups (*p* < 0.001), but the differences between hypertension and preeclampsia did not reach statistical significance (*p* > 0.05).

The data obtained indicate that already at the stage of gestational hypertension, there is activation of cellular (CD8^+^, CD56^+^) and humoral (CD19^+^) immune components associated with a violation of the angiogenic balance. These changes persist with progression to preeclampsia, reflecting the common immunopathogenic mechanisms of vascular complications of pregnancy.

The nonparametric Kruskal–Wallis test was used to assess differences in the level of the CD56^+^TNF^+^ subpopulation between the three clinical groups (control, gestational hypertension, preeclampsia).

According to the Kruskal–Wallis analysis, significant differences in cytokine expression were observed among study groups for both CD56^+^TNF^+^ and CD14^+^IL-10^+^ markers (*p* < 0.001 for both).

Post hoc pairwise comparisons (Dunn–Bonferroni) showed that expression levels were significantly higher in both gestational hypertension and preeclampsia compared to the control group (*p* < 0.001 and *p* = 0.002, respectively), whereas no differences were observed between the hypertensive and preeclamptic groups (*p* > 0.05).

Thus, increased TNF expression in CD56^+^ cells is already observed at the stage of gestational hypertension and persists with the development of preeclampsia, reflecting the activation of inflammatory and cytokine-mediated mechanisms in the pathogenesis of these conditions. The increase in IL-10 expression in monocytes reflects the compensatory activation of anti-inflammatory mechanisms in response to systemic inflammation characteristic of complicated pregnancy.

### 3.4. Receiver Operating Characteristic (ROC) Analysis

We performed a ROC to evaluate the feasibility of using intracellular cytokines to differentiate between patients with gestational hypertension and preeclampsia. The results are presented in Table 5.

**Figure 1:**
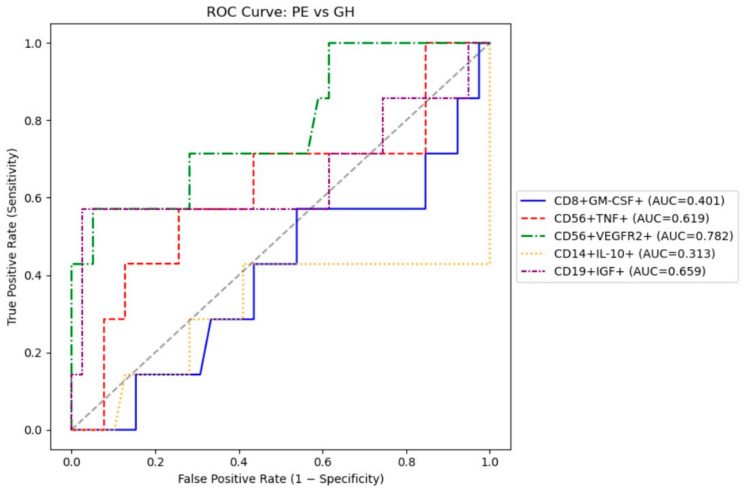
ROC curves for potential markers in discriminating preeclampsia and gestational hypertension.

The most optimal marker for differentiating preeclampsia and gestational hypertension were CD56+VEGFR2+ and CD19+IGF+ with AUC ~0.66–0.78), both achieve moderate 57% sensitivity and good specificity (>95%) (Figure 1).

## 4. Discussion

Our study demonstrated distinct immunological alterations in pregnant women with gestational hypertension (GH) and preeclampsia (PE) compared to healthy controls.

Women with PE showed a significant increase in the proportion of CD8^+^GM-CSF^+^, CD56^+^VEGFR2^+^, CD19^+^IGF^+^, and CD14^+^IL-10^+^ cells, and a decrease in CD56^+^TNF^+^ expression. These findings support the hypothesis of impaired regulation of innate and adaptive immunity at the maternal–fetal interface. The observed imbalance between pro-inflammatory and anti-inflammatory mediators appears to play a central role in the pathogenesis of hypertensive disorders of pregnancy and may serve as a potential source of immunological biomarkers for early disease detection.

Increased expression of GM-CSF^+^ in CD8^+^ cells may reflect activation of the cytotoxic immune system and an enhanced inflammatory response. A significant increase in CD14^+^IL-10^+^ cells level indicates compensatory activation of anti-inflammatory mechanisms; however, judging by the severity of clinical manifestations, this regulation is insufficient to prevent the development of PE. At the same time, a decrease in TNF production in CD56^+^ cells (natural killer cells) may indicate a disruption of their effector function, which can affect placentation processes and control of local inflammation.

TNF-α. TNF-α is known to be a key pro-inflammatory cytokine, the level of which increases in complicated pregnancies, including PE. A number of studies have shown that excessive production of TNF-α in the placenta and peripheral blood is associated with endothelial dysfunction and increased vascular tone [9]. Recent data on the genetic mechanisms regulating TNF-α production are of particular interest. According to a meta-analysis by [17]), which included 32 publications, TNF-α rs1800629 (G/A) polymorphism is significantly associated with an increased risk of developing preeclampsia. Moreover, this association is particularly pronounced in Asian populations (OR up to 2.3 in the dominant model), which emphasizes the ethnic specificity and genetic predisposition to TNF-α hyperproduction in pregnant women [17]. These data suggest that the differences we identified in intracellular TNF-α expression in women from the Almaty cohort may be partly due to genetic factors, including this polymorphism. However, a large cohort study by Adomi et al. (2024), which included more than 4.3 million pregnancies, showed that the use of TNF-α inhibitors in the first and second trimesters does not reduce the risk of early or late preeclampsia (RR 1.25 [0.93–1.67] and RR 0.99 [0.81–1.22], respectively) [18]. This indicates that, despite the pathogenetic significance of TNF-α, systemic blockade of this cytokine does not prevent the development of the disease. Consequently, the identified imbalance should be considered a biomarker of a pathological phenotype rather than a direct therapeutic target.

IL-10. The immunoregulatory cytokine IL-10 plays a central role in maintaining tolerance during pregnancy. Experimental studies have shown that the addition of IL-10 increases the number of Tregs and reduces blood pressure in the RUPP (Reduced Uterine Perfusion Pressure) model in rats [19]. A review by Cubro et al. (2018) highlights its role as a potential therapeutic factor: IL-10 regulates vascular response and enhances cellular interactions at the maternal–fetal interface [20]. According to a meta-analysis by Nath et al. (2020), circulating IL-10 levels do not differ before the onset of PE (SMD ≈ −0.01, *p* = 0.76), but decrease significantly at the time of disease onset (SMD ≈ −0.79, *p* = 0.0004), confirming its role as a potential biomarker of PE activity [21]. Interestingly, our cohort showed an increase in CD14^+^IL-10^+^ cells, which can be interpreted as an attempt at a compensatory anti-inflammatory response.

GM-CSF. GM-CSF plays a key role in regulating the immune response during pregnancy: it stimulates the differentiation of macrophages and dendritic cells and influences the balance of pro-inflammatory and regulatory T cells in the uteroplacental interface. However, this cytokine has been less studied in the context of PE. In the works of Jancsura et al. (2023) and Jancsura et al. (2025) [22,23] showed that GM-CSF levels in overweight women who subsequently developed preeclampsia were significantly elevated as early as the first trimester compared to women without PE. This indicates that GM-CSF may be an early marker of inflammation activation. At the same time, the dynamics differed from the control group: while in healthy women the concentration of inflammatory markers (including GM-CSF) increased as pregnancy progressed, in women with PE there was no such “physiological rise”. This may reflect early depletion or disruption of immune regulation, which creates the conditions for placental dysfunction [22,23].

Our data also revealed an increase in CD8^+^GM-CSF^+^ cells in women with PE. This indicates that T lymphocytes contribute to the hyperinflammatory background of the disease by enhancing GM-CSF production. This finding supports the hypothesis that GM-CSF is not just a systemic serum marker, but also the result of cellular restructuring of the immune response.

Our study showed increased IGF-1 production by B cells in preeclampsia. However, other studies have shown low IGF-1 expression in women with preeclampsia compared to women in normal pregnancy [16]. In patients with preeclampsia, the IGF-1 (insulin-like growth factor 1) expression was significantly reduced compared with healthy pregnancy, associated with a downregulation of ZEB1 [24]. This study also showed increased expression of vascular endothelial growth factor receptor 2 (VEGFR2) by natural killer cells in women with preeclampsia. Pathological angiogenesis is caused by the overactivity of VEGFR-2 that affects tissue perfusion and blood flow regulation [25].

For differentiating PE from GH, CD56+VEGFR2+ and CD19+IGF+ with moderate 57% sensitivity and good specificity (>95%) should be prioritized. Taken together, these findings indicate that preeclampsia and gestational hypertension share a common pattern of immune dysregulation characterized by an imbalance between pro-inflammatory, angiogenic, and regulatory cytokines. However, the predominance of cytotoxic and pro-angiogenic cell activation in preeclampsia suggests a more profound disruption of maternal immune adaptation, which may contribute to the clinical severity of the disease.

## 5. Limitations and Future Direction

This study has several limitations. First, the sample size was small, particularly in the preeclampsia group, which reduces statistical power and limits the generalizability of the findings. Second, the study design was cross-sectional, and therefore causal relationships between intracellular cytokine profiles and the development of preeclampsia cannot be established. Third, only a selected panel of cytokines (TNF, GM-CSF, IGF, IL-10) was analyzed, while other relevant mediators may also play a role. Finally, all participants were recruited from a single clinical center, which may limit external validity. Despite these constraints, the study provides valuable pilot data and highlights directions for future large-scale, multicenter research.

Another limitation is the use of a BD FACS Calibur, which restricted the complexity of multicolor immunophenotyping. Although the antibody panel was carefully optimized to avoid spectral overlap, future studies using high-parameter cytometers would allow more comprehensive profiling of immune cell subsets and cytokine expression patterns.

## 6. Conclusions

Preeclampsia and gestational hypertension remain among the most significant complications of pregnancy, contributing substantially to maternal and perinatal morbidity. Analysis of intracellular cytokine expression demonstrated that women with gestational hypertension and preeclampsia exhibit pronounced activation of immune cells producing growth and angiogenic factors, particularly GM-CSF, IGF^+^, and VEGFR2^+^. These changes were accompanied by an increase in anti-inflammatory IL-10 monocytes, suggesting a compensatory regulatory response.

The observed imbalance between pro-inflammatory, angiogenic, and regulatory cytokine-producing cells indicates immune dysregulation at the maternal–fetal interface, which may underlie impaired vascular adaptation and placental dysfunction characteristic of hypertensive disorders of pregnancy. While both GH and PE groups showed similar immunophenotypic shifts compared to the control group, the alterations were more pronounced in preeclampsia, reflecting its greater clinical severity.

These findings highlight the potential diagnostic value of intracellular cytokine profiling for distinguishing the immune signatures of gestational hypertension and preeclampsia. However, given the limited sample size, the results should be interpreted with caution and warrant validation in larger, multicenter studies integrating functional and longitudinal assessments.

## Figures and Tables

**Table 1:** Clinical data and pregnancy outcomes of the GH, PE and control groups.

Indicators	Control (n = 32)	95% CI	GH (n = 39)	95% CI	PE (n = 7)	95% CI	*p*
Age	33.3 ± 6.39	(31; 35.7)	34.5 ± 6.8	(32.3; 36.6)	33.5 ± 3.86	(29.9; 37.1)	0.665
Weight	81.3 ± 14.9	(75.5; 86.8)	86.6 ± 18.3	(80.8; 92.4)	82.1 ± 9.37	(73.4; 90.8)	0.06
Height	163 ± 5.7	(161; 165)	158.2 ± 24.4	(150.5; 165.9)	166 ± 1.5	(164.3; 167)	0.109
Blood pressure							
systolic	102 ± 9.57	(80; 120)	142.1 ± 8	(139.5; 144.6)	140 ± 11	(129.1; 149.4)	<0.001
diastolic	67.5 ± 12.4	(60; 120)	90.4 ± 7.2	(88.4; 92.7)	84.3 ± 7.87	(77; 91.5)	<0.001
Gest. age at diagnosis	-	-	30.7 ± 6.7	(28.6; 32.8)	36.2 ± 2.14	(34.1 ± 40.1)	0.06
Gestation period	39.5 ± 1.21	(39; 40)	38.5 ± 0.8	(38.2; 38.7)	36.5 ± 4	(32.8; 40.2)	<0.001
Child’s weight in g	3600 ± 420	(3443; 3756)	3428 ± 475	(3277; 3578)	3041 ± 672	(2418; 3662)	0.05

**Table 2:** Descriptive analysis of immunological parameters.

Double Phenotyping Markers	Control (n = 32)		GH (n = 39)		PE (n = 7)	
	Mean	CI 95%/IQR	Mean	CI 95%/IQR	Mean	CI 95%/IQR
CD8^+^GM-CSF^+^	7.33	(6.74–7.83)	29.97	(28.77–31.0)	28.58	(25.04–31.9)
CD56^+^TNF^+^	15.95	[1.84]	1.42	[1.06]	2.19	[2.31]
CD56^+^VEGFR2^+^	0.55	(0.39–0.78)	10.53	(9.97–11.28)	14.2	(11.02–17.43)
CD14^+^IL-10^+^	0.77	[1.09]	68.65	[7.52]	55.68	[31.02]
CD19^+^IGF^+^	1.08	(0.94–1.26)	9.68	(8.98–10.39)	12.16	(8.34–15.72)

**Table 3:** The difference between the three groups (one-way analysis of variance (ANOVA) of cytokine-producing cell populations between women with gestational hypertension (GH), preeclampsia (PE), and healthy controls.

Marker	Comparison	Mean Difference (I–J)	*p*-Value	Interpretation
CD8^+^GM-CSF^+^	Control vs. GH	−22.60	<0.001	↑ in GH
	Control vs. PE	−21.19	<0.001	↑ in PE
	GH vs. PE	1.41	0.633	n.c.
CD56^+^VEGFR2^+^	Control vs. GH	−10.03	<0.001	↑ in GH
	Control vs. PE	−13.63	<0.001	↑ in PE
	GH vs. PE	−3.59	0.075	n.c.
CD19^+^IGF^+^	Control vs. GH	−8.59	<0.001	↑ in GH
	Control vs. PE	−10.93	<0.001	↑ in PE
	GH vs. PE	−2.34	0.344	n.c.

↑—increase, n.c.—no changes.

**Table 4:** Post hoc multiple comparisons (Games–Howell test) of cytokine-producing cell populations between women with gestational hypertension (GH), preeclampsia (PE), and healthy controls.

Marker	Comparison	Test Statistic	Adjusted *p*-Value	Interpretation
CD56^+^TNF^+^	GH vs. PE	−5.48	1.000	n.c.
	GH vs. Control	39.83	<0.001	↑ in GH
	PE vs. Control	34.36	0.001	↑ in PE
CD14^+^IL-10^+^	GH vs. PE	8.59	1.000	n.c.
	GH vs. Control	40.31	<0.001	↑ in GH
	PE vs. Control	31.71	0.002	↑ in PE

↑—increase, n.c.—no changes.

**Table 5:** ROC analysis results.

Potential Markers	AUC	Sensitivity	Specificity
CD8^+^GM-CSF^+^	0.40	0.57	0.46
CD14^+^IL10^+^	0.31	0.43	0.59
CD56^+^TNF^+^	0.62	0.57	0.74
CD56^+^VEGFR2^+^	0.78	0.57	0.95
CD19^+^IGF^+^	0.66	0.57	0.97

## Data Availability

The data presented in this study are available on request from the corresponding author. The data are not publicly available due to privacy and ethical issues.

## Supplementary Materials

The following supporting information can be downloaded at: https://www.mdpi.com/article/10.3390/biomedicines13123122/s1, Figure S1: STROBE-compliant participant flow diagram.

## Abbreviations

The following abbreviations are used in this manuscript:

PE	Preeclampsia
BP	blood pressure
ISSHP	International Society for the Study of Hypertension in Pregnancy
GM-CSF	Granulocyte-Macrophage Colony-Stimulating Factor
TNF-α	Tumor Necrosis Factor alpha
CI	Confidence Intervals
BD	Becton Dickinson
FACS	Fluorescence-Activated Cell Sorting
