# Supplementary figures and images for: Blood Immunophenotyping in Prediction of Gestational Hypertensive Conditions
Source: Biomedicines. 2025 Dec 18;13(12):3122. doi: 10.3390/biomedicines13123122 (PMC12730196; doi:10.3390/biomedicines13123122)

**Figure S1. STROBE-compliant participant flow diagram**

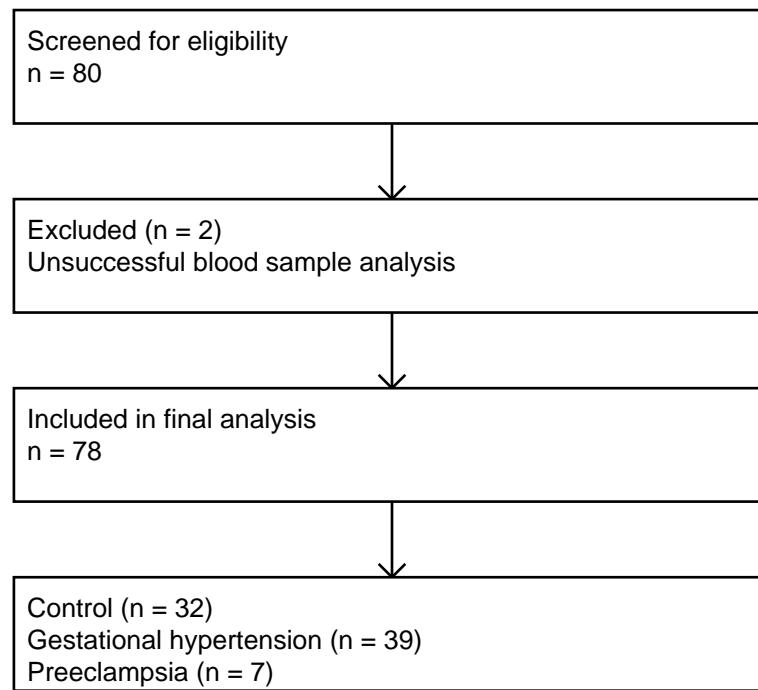

Supplement: Supplementary file 1 [file biomedicines-13-03122-s001.zip › biomedicines-3987833-supplementary.pdf]
